# Decreased Antibiotic Susceptibility Driven by Global Remodeling of the *Klebsiella pneumoniae* Proteome*[Fn FN2]

**DOI:** 10.1074/mcp.RA118.000739

**Published:** 2019-01-07

**Authors:** Sarah L. Keasey, Moo-Jin Suh, Sudipto Das, Candace D. Blancett, Xiankun Zeng, Thorkell Andresson, Mei G. Sun, Robert G. Ulrich

**Affiliations:** From the ‡Department of Biological Sciences, University of Maryland Baltimore County, Baltimore, Maryland;; §Molecular and Translational Sciences Division, U.S. Army Medical Research Institute of Infectious Diseases, Frederick, Maryland;; ¶Laboratory of Proteomics and Analytical Technologies, Leidos Biomedical Research, Frederick National Laboratory for Cancer Research, NCI-Frederick, Frederick, Maryland;; ‖Pathology Division, U.S. Army Medical Research Institute of Infectious Diseases, Frederick, Maryland

**Keywords:** Bacteria, Drug resistance, Mass Spectrometry, Systems biology*, Pathogens

## Abstract

Bacteria can circumvent the effect of antibiotics by transitioning to a poorly understood physiological state that does not involve conventional genetic elements of resistance. Here we examine antibiotic susceptibility with a Class A β-lactamase+ invasive strain of *Klebsiella pneumoniae* that was isolated from a lethal outbreak within laboratory colonies of *Chlorocebus aethiops sabaeus* monkeys. Bacterial responses to the ribosomal synthesis inhibitors streptomycin and doxycycline resulted in distinct proteomic adjustments that facilitated decreased susceptibility to each antibiotic. Drug-specific changes to proteomes included proteins for receptor-mediated membrane transport and sugar utilization, central metabolism, and capsule production, whereas mechanisms common to both antibiotics included elevated scavenging of reactive oxygen species and turnover of misfolded proteins. Resistance to combined antibiotics presented integrated adjustments to protein levels as well as unique drug-specific proteomic features. Our results demonstrate that dampening of *Klebsiella pneumoniae* susceptibility involves global remodeling of the bacterial proteome to counter the effects of antibiotics and stabilize growth.

## Abstract (precis)

Keasey *et al*. investigate the proteomic mechanisms used to circumvent the effect of antibiotics by Gram-negative bacteria that do not harbor traditional genetic elements of resistance. The proteomes of *Klebsiella pneumoniae* bacteria that survived treatment with antibiotic inhibitors of ribosomal protein synthesis were examined by label-free quantitative mass spectrometry. Taking a systems approach, they identify both common and drug-specific molecular pathways that facilitate decreased susceptibility to antibiotics. These results explain why some antibiotic treatments fail even with sensitive pathogens.

## Graphical Abstract

**Figure:**
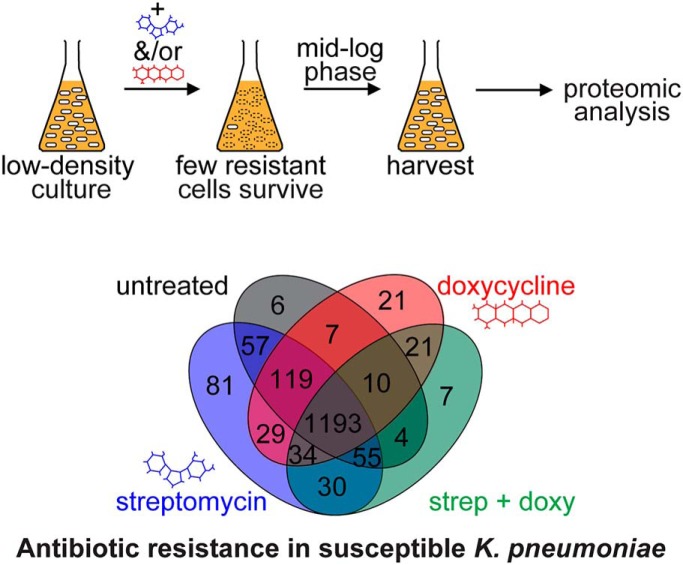

**Highlights**

- Global proteomic remodeling alters antibiotic susceptibility in *K. pneumoniae*.
- Drug specific transport, sugar utilization, central metabolism, capsule synthesis.
- Common pathways of reactive oxygen scavenging, turnover of misfolded proteins.
- Integrated adjustments and unique drug-specific features for drug combinations.

As a commensal Gram-negative bacterium that colonizes mucosal surfaces of the gastrointestinal and respiratory tracts, *K. pneumoniae* readily adheres to medical devices, thereby increasing risk of infection for patients undergoing procedures with reused or inserted medical equipment (1, 2), and immunocompromised individuals are especially vulnerable (3). The emergence of more-virulent and invasive *K. pneumoniae* strains (4) has broadened the susceptible population to include individuals who are healthy and non-immunocompromised. Moreover, the failure of first-line broad-spectrum therapies against clinical isolates is leading to an increase of infections that are chronic and recalcitrant to treatment (3). Recently, an outbreak of carbapenem-resistant *K. pneumoniae* occurred at the Clinical Center of the U.S. National Institutes of Health, causing the deaths of 11 of 18 affected patients (3). Moreover, an outbreak of chronic and lethal infections that occurred within laboratory primate colonies of *Chlorocebus aethiops sabaeus* (5, 6) was caused by a hypermucoviscous and invasive strain of *K. pneumoniae V513* (*KpV513*)^1^. Animals infected by the *KpV513* strain did not respond to antibiotics, and this outbreak provides a primate model to better understand drug interactions with this emerging bacterial pathogen.

Bacteria can survive antibiotic treatment through acquisition of genetically encoded elements that confer specific resistance or by transitioning to a phenotypic state of resistance that can be experimentally demonstrated by culturing with antibiotic levels that are below the minimum inhibitory concentration (sub-MIC). Further, the termination of antibiotic therapy for chronic infections (7–9) often results in disease relapse because of the resurgent growth of bacteria that survived antibiotic exposure. Previous studies have reported that susceptible Gram-negative bacteria that survive antibiotic treatment exhibit stochastic variations in levels of ppGpp (10) and ATP (11) that coincide with slow growth or states of dormancy. However, many factors must come into play for infection to continue after antibiotic treatment, and it is possible that additional features can be detected by proteomic-level studies of antibiotic responses. Here we examined population and proteomic dynamics of *KpV513* under experimental conditions that replicate phenotypic resistance to disparate classes of drugs.

## MATERIALS AND METHODS

### 

#### 

##### Microscopy

For immunohistochemistry, formalin-fixed and paraffin embedded (FFPE) tissue sections were de-paraffinized using xylene and a series of ethanol washes. The sections were treated with endogenous peroxide and non-specific antibody blocking reagents, and incubated with rabbit anti- *K. pneumoniae* polyclonal antibody (1:2000, Thermofisher Scientific, Waltham, MA) for 2 h at room temperature. Sections were visualized using a horseradish peroxidase-labeled polymer, Envision + system (anti-rabbit) (Agilent, Santa Clara, CA) subjected to reaction with the chromogen diaminobenzidine. For immunofluorescence, formalin-fixed and paraffin embedded tissue sections were deparaffinized using xylene and a series of ethanol washes. After 0.1% Sudan black B (Sigma-Aldrich, St. Louis, MO) treatment to eliminate autofluorescent backgrounds, the sections were heated in citrate buffer (pH 6.0) for 15 min to reverse formaldehyde crosslinks. After rinses with PBS (pH 7.4), the sections were blocked with PBS containing 5% normal goat serum overnight at 4 °C, and incubated with rabbit anti- *K. pneumoniae* polyclonal antibody (1:2000, Thermofisher Scientific) for 2 h at room temperature. After rinses with PBS, the sections were incubated with secondary Alexa Fluor 568 conjugated goat anti-rabbit antibody for 1 h at room temperature. Sections were cover slipped using Vectashield mounting medium with DAPI (Vector Laboratories, Burlingame, CA). Images were captured on a Zeiss LSM 780 confocal system (Carl Zeiss Microscopy, Jena, Germany) and processed using ImageJ software.

##### Electron Microscopy

Bacteria were grown to mid-log phase (OD_600_ = 0.5) in streptomycin, doxycycline or untreated control medium. The cells were harvested by centrifugation at 5000 × *g* for 10 min at 4 °C. Bacteria were fixed at room temperature for 1 h in EM primary fixatives of 2.5% formaldehyde and 2.5% glutaraldehyde in 0.1 m sodium cacodylate (pH 7.4) buffer. For transmission electron microscopy, the fixed bacteria were washed three times for 10 min each in 0.1 m sodium cacodylate buffer, incubated for 1 h in 1% osmium tetroxide in 0.1 m sodium cacodylate buffer, and washed with distilled water for 10 min. The samples were stained for 1 h in 1% uranyl acetate, dehydrated in an ethanol series of 30%, 50%, 75%, and 95%, successively, for 10 min each, followed by three 10 min washes in 100% ethanol and two 10 min washes in propylene oxide. Samples were infiltrated with agitation at room temperature in well-mixed 50% v/v propylene oxide/Epon 812 (Electron Microscopy Sciences, Hatfield, PA, Cat#RT14120) for 1 h, followed by 100% Epon 812 three times for 1 h each. The samples were placed in an oven and allowed to polymerize at 60 °C for 24 h. Thin sections (∼80 nm) were collected and pre-stained with 1% uranyl acetate and Sato lead before examination in a JEOL 1011 (Joel USA, Inc., Peabody, MA) transmission electron microscope at 80kV. Digital images were acquired using AMT camera system (Advanced Microscopy Techniques, Corporation, Woburn, PA). For scanning electron microscopy, the fixed bacteria were transferred to filter membranes under vacuum, and washed and dehydrated as described above, excluding the propylene oxide wash steps. The prepared samples were transferred to a critical point dryer to reach critical point dryness, mounted on specimen stubs, and sputter coated with 6 nm iridium. The samples were loaded into Zeiss Sigma field emission scanning electron microscope (Carl Zeiss Microscopy) using 2kV InLens secondary electron detector, and digital images were acquired using SmartSEM software. Two biological replicates were performed for each condition.

##### Bacterial Cultures and Antimicrobial Susceptibility Testing

Bacterial cultures were grown at 37 °C with 250 rpm shaking in Luria-Bertani (*KpV513,* clinical isolates of *K. pneumoniae, Escherichia coli, Acinetobacter baumannii*) or heart infusion (*Yersinia pestis*) broth with aeration. After 16h, bacteria were diluted 1:100 in culture broth and grown to the mid-log phase (OD_600_ ∼ 0.5) twice to synchronize growth. Plating of serially diluted cultures established that 5 × 10^8^ colony forming units (cfu) were present in mid-log phase cultures. Determination of antibiotic minimum inhibitory concentrations (MIC) was performed as previously described (12), in microplate format. Absorbance of each microplate well (200 μl culture) at 600 nm was monitored using a Bioscreen CTM Automated Microbiology Growth Curve Analysis System (Growth Curves USA, Piscataway NJ). The MIC end point was determined as the lowest concentration of antibiotic at which there was no visible growth after 20 h. For isolation of untreated and antibiotic treated cells, replicate wells (*n* = 6) of *KpV513* bacteria in culture ± antibiotic at 50% of the MIC concentration (MIC_50_) were harvested at mid-log phase by centrifugation at 5000 × *g* for 10 min at 4 °C. Supernatant absorbance was recorded and cell pellets were stored at −80 °C. Six biological replicates were performed for each condition, except for the combination treatment of streptomycin + doxycycline, which was performed in triplicate. Using the string test on doxycycline-treated colonies, which exhibited a distinct mucoid phenotype, hypermucoviscosity was defined by formation of viscous strings > 5 mm long (13). For cyclic administration of antibiotics, untreated or antibiotic treated *KpV513* were treated at a low level of detection (*i.e.* at their calculated *C_t_*, see below) with MIC_50_ antibiotics.

##### Acid Production and Glucose Utilization

*K. pneumoniae* were cultured untreated or with antibiotic(s) at the MIC_50_, harvested during mid-exponential growth, and centrifuged at 5000 × *g* for 10 min to pellet cells. The pH indicator bromthymol blue (0.03%) was added to culture supernatants (*n* = 3), and absorbance at 620 nm (blue, pH ≥ 7.2) and 425 nm (yellow, pH ≤ 6), which are peak absorption wavelengths of the deprotonated and protonated forms of bromthymol blue, respectively, were recorded. Glucose utilization was assessed under hypoxic and aerobic conditions using Hugh and Leifson's oxidation fermentation basal medium (pH 7.1) with glucose as the sole carbohydrate source. Cell pellets were washed twice in antibiotic-free broth and inoculated as stabs into two tubes of oxidative fermentative medium. One tube from each pair was overlaid with mineral oil as a barrier to oxygen diffusion (hypoxic conditions). Tubes were incubated at 37 °C for 5 h and levels of acid production from glucose metabolism were recorded as the percentage of medium within the tube that changed color from blue (pH 7.1) to yellow (pH 6).

##### Growth Curve Analysis

The time to detectable growth, *C_t_*, was calculated by fitting an exponential curve to the initial phase of growth and solving for *x* at *y* = 10^−6^. For predictions using the discrete dynamical systems model, measurements of bacterial population density are denoted by *D*, where *D_o_* is the starting cell density and *D_t_* reflects bacterial density at subsequent time points *t*. Population density was recorded every 15 min, and *t* denotes rescaled time, such that each 15 min interval represents one unit of time (*t* = 0, 1, 2, 3, …; for *t* = 3, the corresponding time in the experiment is 3 × 15 min = 45 min). The change in population density (*D*_*t*+1_ − *D_t_*) with time ((*t*+1) − *t*) exhibited a parabolic pattern, in which the population change increased faster than linearly, indicating that the doubling rate of bacteria was greater than the measurement time interval (15 min). Assuming constant rates of cellular division, we expect the population change (*D*_*t*+1_ − *D_t_*) to be a fixed fraction of the population *Dt*, and the model should be of the form

(Eq. 1) $$D_{t+1}-D_{t}=rD_{t}$$

where *r* is a constant that reflects the proportion of cells dividing during each time interval. We plotted population density *versus* population change for each time interval during the early phase of detectable growth. The resulting linear line intersects the origin and the slope of the line gives *r*. Here, 15/*r* yields the dividing time in minutes. In function form, (Eq.1) becomes

(Eq. 2) $$f\left(D_{t}\right)=\left(1/r\right)\times D_{t}=D_{t+1}$$

and iterating *f*, we can express the general solution of the discrete dynamical system for the initial condition *D_o_* as

(Eq. 3) $$D_{t}=D_{o}\times {\left(1/r\right)}^{t}$$

To estimate the proportion of cells that initiated resurgent growth, *D_o_* of untreated *KpV513* was calculated using (Eq.3). Because *KpV513* cultures were serially diluted such that ∼200cfu were inoculated into each microwell, we validated our mathematical model by comparing the theoretical *D_o_* to the number of cfu experimentally added to culture. Over replicate experiments (*n* = 6), *D_o_* = 200 ± 25cfu. Next, because antibiotic-treated *KpV513* cultures were inoculated with the same stock of serially diluted cells, we used (Eq. 3) to estimate the theoretical *D_t_* of treated cultures at the untreated *C_t_*, using *r* from growth of treated cells and *D_o_* from untreated cultures. Finally, again using (Eq.3), we could estimate *D_o_* of treated cultures based on the treated theoretical *D_t_* and *r*.

To describe the effect of decreasing growth rates on culture capacity, we fit a logistic model to measurements of culture absorbance approaching stationary phase. The relative population change was reduced as population size increases, eventually approaching zero as the environmental carrying capacity was reached. Therefore, the relative population change was proportional to the unused carrying capacity, such that

(Eq. 4) $$\frac{D_{t+1}-D_{t}=r}{D_{t}}\frac{\left(1-D_{t}\right)}{M}$$

For decaying rates of growth, we plotted the relative population change (*D*_*t*+1_ − *D_t_*)/*D_t_ versus* the population size *D_t_*. If x = *D_t_* and y = (*D*_*t*+1_ − *D_t_*)/*D_t_*, then

(Eq. 5) y=r(1−x/M)=r−(r/M)x

and the data should lie along a line with slope –*r/M* and y-intercept of *r*. We calculated *M*, which reflects *M_initial_* because it is based on the maximum capacity achieved prior to declining growth rates, and compared it to *M_decay_*, which is the experimentally observed maximum culture density.

Growth curves were derived and integrated for phase-portrait graphs (Fig. 4) using Origin Pro Software (OriginLab Corporation, Northampton, MA).

##### Imaging Mass Spectrometry

MALDI imaging MS was performed using a Solarix dual source Fourier transform-ion cyclotron resonance (FT-ICR) mass spectrometer (Bruker Daltonics, Billerica, MA) with a SmartBeam II laser operating at 1kHz, a laser spot size of 25 μm, and a raster width of 200 μm for general profiling. Spectra in positive ion mode were generated using 100 laser shots at a frequency of 1 kHz. Ion images for tissue sections were acquired in 4 h at a peak resolution of ∼125,000 (FWHM, *m*/*z* 400). Following MS analysis, data was loaded into FlexImaging Software focusing on the *m*/*z* = 700–3500 range and reduced to 0.98 ICR Reduction Noise thresholds. All data were normalized by root mean squares.

##### Sample Preparation for Shotgun Proteomics

*KpV513* cell pellets were suspended and sonicated for 200 s (10 s on, 10 s off, 10 runs at 20W) in an ice bath of 1 mL ice-cold 25 mm Tris-HCl (pH 7.4, added lysozyme, DNase I, Protease Inhibitor). Sonicated bacterial suspensions were centrifuged at 5000 × *g* at 10 °C for 20 min. Supernatants were analyzed by SDS-PAGE (Bio-Rad, Hercules, CA) followed by Coomassie stain, and total protein concentration was determined using the Bradford protein assay (Bio-Rad). 100 μg of total cell lysate was used to generate tryptic digest using the “filter-aided sample preparation” (FASP) protein digestion method (14). *KpV513* proteins in buffer (50 mm ammonium bicarbonate, 8 m urea) were reduced by dithiothretiol and alkylated with 20 mm iodoacetamide in the dark for 1 h. After diluting into 1 m urea, alkylated proteins were digested with trypsin (1:100 w/w, Promega, Madison, WI) at 37 °C for 12 h and cleaned with Stage-Tip prior to 1D-LC-MS/MS (15).

##### Liquid Chromatography and Tandem Mass Spectrometry

All LC-MS/MS analysis was performed on both Orbitrap Elite and Fusion Classic mass spectrometers (Thermo Scientific, San Jose, CA). 100 ng of desalted tryptic protein digest was loaded onto Acclaim™ PepMap™ 100 C_18_ LC column (Thermo Scientific) using a Thermo Easy nLC 1000 LC system (Thermo Scientific) connected online with Orbitrap Fusion Classic mass spectrometer. After sample injection, the peptides were eluted using LC methods, running 1% to 35% LC gradients of aqueous, formic acid/acidified acetonitrile over 100 min at a flow rate of 300 nl/min. The Fusion mass spectrometer was operated in a data dependent mode in which each full MS scan was followed by 20 data-dependent MS/MS scans. Voltage setting at the nano-spray was set at 2.3 kV for stable spray. Each MS^1^ scan was performed in the Orbitrap at a resolution of 60,000, with maximum injection time of 50 ms and AGC target set at 2 × 10^5^ counts. For MS^2^ scan, a normalized Collison energy setting of 29 was used with a maximum injection time of 75 ms and AGC target set to 1 × 10^4^ counts.

For Orbitrap Elite MS, the desalted tryptic protein digest was loaded onto an Ultimate 3000 HPLC (Thermo Fisher Scientific) with a pulled-tip 75 μm × 15 cm C_18_ column (New Objective, PicoFrit column) at a flow rate of 300 nl/min. The peptides were eluted using LC methods as described above. Voltage settings at the nano-spray source were optimized at 2.0 kV to ensure stable fine spray. The cycles of 10 data-dependent MS/MS scans per MS^1^ scan were performed. For all collision-induced dissociation (CID) scans, a normalized collision energy of 30 was used, the maximum inject time was 100 ms and maximum ion counts sat to 1 × 10^4^ counts for MS^2^. Two technical replicates were performed for each biological replicate. Two biological samples were also tested for comparison of data between two mass spectrometers (Fusion and Elite).

##### Protein Identification and Quantification

Raw MS data were collected and analyzed using Proteome Discoverer (version 2.2, Thermo Scientific) with Sequest HT search software and MaxQuant (version 1.6.0.1) incorporated with the Andromeda search engine. Each search engine was supplied with a database comprising comprehensive *K. pneumoniae* protein sequences (downloaded on 9 July 2016, 5126 proteins, Taxonomy 272620) combined with common contaminants proteins. Mass tolerance settings were as follows: fragment ion mass 0.6 Da, parent ion tolerance 10 ppm (Sequest); precursor ion mass 10 ppm and fragment ion mass 20 ppm (MaxQuant). Search settings included trypsin as the digestion enzyme, minimal peptide length of six amino acids, and a maximum of two missed cleavage sites. Carbamidomethylation of cysteine was set as a fixed modification, and N-terminal protein acetylation and methionine oxidation as variable modifications. The maximum false discovery rate (FDR) was set at 1%. Two unique peptides were selected for a protein identification using Sequest. This approach was used in initial analysis stages to ensure that raw data sets had enough proteome depth. Combining data files representing two LC-MS/MS replicates for each biological replicate, the label-free quantitation (LFQ) values derived from the LFQ algorithm in MaxQuant were used for quantitative analysis. Default settings for quantification based on MS peak integration and for normalization among all datasets were accepted. Protein isoforms that could not be discriminated based on unique peptides were reported as a single protein group. Proteins not present in at least 15% of the samples were removed. We used abundance distribution-based imputation (ADI) to replace missing values (16, 17). ADI is a randomized imputation method based on normal abundance distribution to simulate a typical abundance region for missing values if experimentally measured (supplemental File S4).

##### Experimental Design and Statistical Rationale

For statistical analyses, proteomic data were used from six biological replicates of LC-MS/MS analyses for each experimental group except the combination treatment of streptomycin + doxycycline, which was performed in triplicate. A non-parametric (Wilcoxon-Mann-Whitney) test was used to distinguish statistically significant changes between antibiotic-treated and the untreated control *KpV513* cultures. Proteins with *p* value < 0.05 and ± 2-fold change were considered significant and differentially abundant. A principal component analysis (PCA) was performed in Perseus software, using a minimum peptide cutoff = 20, only proteins present in at least 80% of the spot maps, and a Benjamini-Hochberg procedure with a significance level of 0.05. Differentially abundant proteins in the proteomes of streptomycin- or doxycycline-treated *KpV513* were mapped to cellular pathways using the KEGG database (www.genome.jp/kegg/pathway.html).

## RESULTS

### 

#### 

##### Culture Model of Antibiotic Resistance

**Fig. 1.**
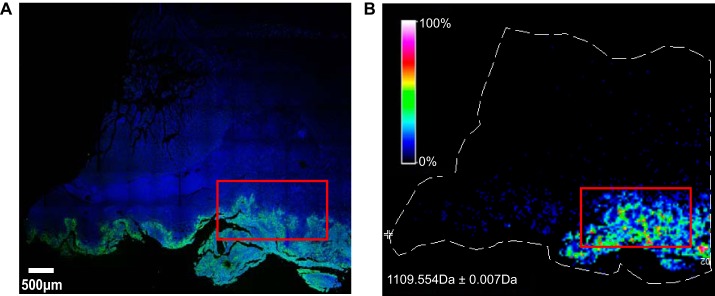
***Klebsiella pneumoniae* infection in the large intestine of a *Chlorocebus aethiops sabaeus* monkey.** Evaluation of cecum tissue procured during necropsy of a subject that presented multiple abscesses of the gastrointestinal tract and succumbed to *K. pneumoniae* infection. *A,* Immunofluorescence to detect nuclei (blue) and *K. pneumoniae* cells (green). *B*, Collection of ion intensity for the *K. pneumoniae* peptide at *m/z* 1109.554 Da from imaging mass spectrometry. The peptide from aminotransferase ArgD protein co-localized with *K. pneumoniae* cells detected by immunofluorescence in (*A*).

The *KpV513* isolate, which was a K2 capsular serotype, caused a severe multisystem disease that was unresponsive to cefazolin antimicrobial therapy. The infections reoccurred within the colony, suggesting a chronic carrier state for one or more of the individual animals. We examined tissue samples procured from an animal that presented multiple abscesses of the gastrointestinal tract and succumbed to infection by *KpV513*. Microscopic evaluation of cecum tissue revealed a significant bacterial burden (Fig. 1*A*) that was accompanied by the presence of numerous infiltrating macrophages (supplemental Fig. S1). Ion intensity maps of infected cecum tissue, acquired by imaging mass spectrometry, exhibited marked increases in bacterial proteins that co-localized with *KpV513* observed microscopically (Fig. 1*B* and supplemental Fig. S2). For example, the peptide at *m*/*z* = 1109.554 (TLVQSTFADK) was detected from the acetylornithine/succinyldiaminopimelate aminotransferase ArgD of *KpV513*. The bacteria isolated from the infections exhibited a drug resistance profile (supplemental Table S1) comparable to clinical extended-spectrum-beta-lactamase (ESBL)-*K. pneumoniae* isolates that are associated with fatal human infections. Mass spectrometry (supplemental Table S2, S3) and genome sequencing (19) identified a Class A β-lactamase that was harbored by *KpV513*, which confirmed an ESBL designation for this strain.

**Fig. 2.**
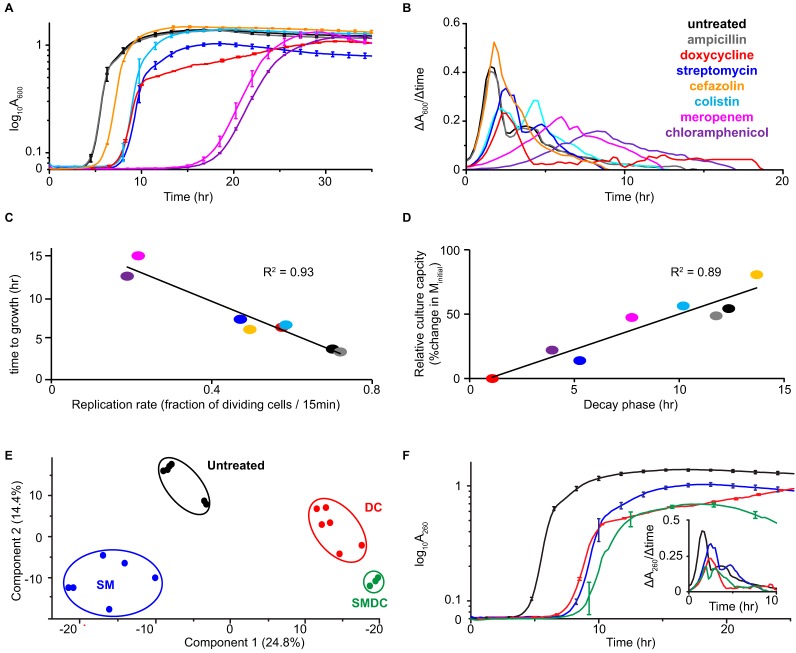
**Growth of *Klebsiella pneumoniae* in response to antibiotics that target cell wall synthesis or ribosomal function.** Extended spectrum β-lactamase-producing *K. pneumoniae* were cultured untreated (black), with ampicillin (gray) at the highest tested dose (128 μg/ml), or in the presence of susceptible antibiotics at the MIC_50_. *A*, Culture growth curves and (*B*) rates of replication based on first derivative growth curves, overlaid from the time of detectable increases in culture absorbance. *C*, Relationship between the initial rate of replication and time to detectable increase in optical density in early culture. *D*, Relationship between culture density accumulated during the decay phase of growth and the relative culture capacity that was normalized to the maximum capacity attained at the highest replication rates during the initial phase of growth (*M_initial_*). *E*, Principal component analysis of proteins identified by mass spectrometry, for all biological replicates (closed circles) of each treatment condition: streptomycin, SM; doxycycline, DC; combination of SM and DC, SMDC; untreated. *F*, Culture growth and replication rates (inset) of bacteria tolerant to ribosomal synthesis inhibitors. Coloring is consistent throughout: cefazolin, orange; meropenem, pink; colistin, cyan; streptomycin, blue; doxycycline, red; chloramphenicol, purple; combination of streptomycin and doxycycline, green.

We developed a culture model with *KpV513* to further examine responses of this invasive strain to antibiotics. In general, the expansion of bacterial populations can be described by four sequential phases: a lag with no detectable change (*C_t_*), the initial highest rate of expansion, a decaying rate of expansion, and a stationary phase of growth equilibrium that is limited by the carrying capacity of the culture environment. Because the minimum inhibitory concentration (MIC) represents the drug level that completely blocks growth, 50% of the antibiotic MIC (MIC_50_) was used in broth cultures (Table I). The reduced susceptibility to the penam (ampicillin) and cephem (cefazolin) β-lactams, as well as colistin, was obvious from growth attributes and MICs (Table I). Interestingly, the growth curves for responses to each non-β-lactam antibiotic demonstrated specific values for lengthened *C_t_*, extended time to mid-exponential growth, and maximum cell density (Fig. 2*A*, 2*B*). Similar deviations from normal growth characteristics were also evident in the growth curves of clinical isolates of *E. coli*, *K. pneumoniae*, and *Acinetobacter baumannii*, as well as an attenuated strain of *Yersinia pestis*, that were treated with antibiotics at the MIC_50_ (supplemental Table S1 and supplemental Fig. S3). These results indicated that altered growth kinetics are a common response to antibiotics by Gram-negative bacteria and are not specific to only *KpV513*. A discrete dynamical system method (20) was used to further examine the population responses of *KpV513* to antibiotics. For the initial phase of detectable growth in culture, we noted a decrease (39% ± 8.7%) in the rate of cell division with antibiotic treatment that directly correlated (R^2^ = 0.95) with *C_t_* (Fig. 2*C*) and estimated that ∼ 1.5% (1.4% ± 0.36%) of total bacteria seeded tolerant cultures, regardless of the specific antibiotic. We observed no difference in cell size between untreated and antibiotic-treated *KpV513* by transmission electron microscopy (data not shown), indicating that the results represented actual population increases. Sampling cell density during the initial highest rate of expansion (*M_initial_*) and the decaying rate of expansion (*M_decay_*) provided an indication of the relationship between growth rate and the carrying capacity, which was influenced by the metabolic efficiency of carbon source conversions to biomass and the changing culture environment (pH, temperature, accumulation of toxic products, etc.) (21). Compared with untreated bacteria, antibiotic-treated cells exhibited shortened phases of growth rate decay that directly correlated (R^2^ = 0.89) with reduced carrying capacity, indicating that antibiotic treatment reduced nutrient-to-biomass conversion efficiencies. For example, colistin treatment resulted in 10.4 h of growth-rate decay, which contributed to an additional population expansion that exceeded *M_initial_* by > 50%, whereas cultures treated with doxycycline exhibited a negligible growth decay phase that did not expand past *M_initial_* (Table I, Fig. 2*D*). Antibiotic-treated cells that were cultured with fresh antibiotic-free media reverted to the original drug susceptibility profiles (data not shown).

**Table I:** Growth response of Klebsiella pneumoniae to antibiotic treatment

Treatment^a^	Class	Mechanism Bacteri-	Target	MIC (μg/ml)	Lag (Ct, hr)	Doubling time (min)	*M_initial_* (A_260_)	*M_decay_* (A_260_)	Growth decline (hr)
Untreated					3.71	21.40	0.86	1.32	12
Ampicillin*	Penicillin	cidal	Cell wall	>128	3.37	21.79	0.87	1.29	12
Cefazolin*	Cephalosporin	cidal	Cell wall	4	4.20	30.61	0.77	1.39	14.2
Meropenem	Carbapenem	cidal	Cell wall	1	15.16	69.25	0.85	1.25	11.1
Colistin*	Polymyxin	cidal	Cell wall	4	6.65	25.65	0.85	1.32	10.4
Streptomycin	Aminoglycoside	cidal	30S ribosome	4	7.34	31.84	0.99	1.13	6.5
Doxycycline	Tetracycline	ostatic	30S ribosome	1	6.35	26.08	2.40	1.00	14.8
Chloramphenicol*	Amphenicol	ostatic	50S ribosome	16	12.58	79.24	0.93	1.14	8.5

^a^Growth properties determined at the antibiotic MIC_50_.

*MIC indicates resistance (ampicillin) and reduced susceptibility (cefazolin, colistin, chloramphenicol).

##### Drug-specific Mechanisms of Resistance

**Fig. 3.**
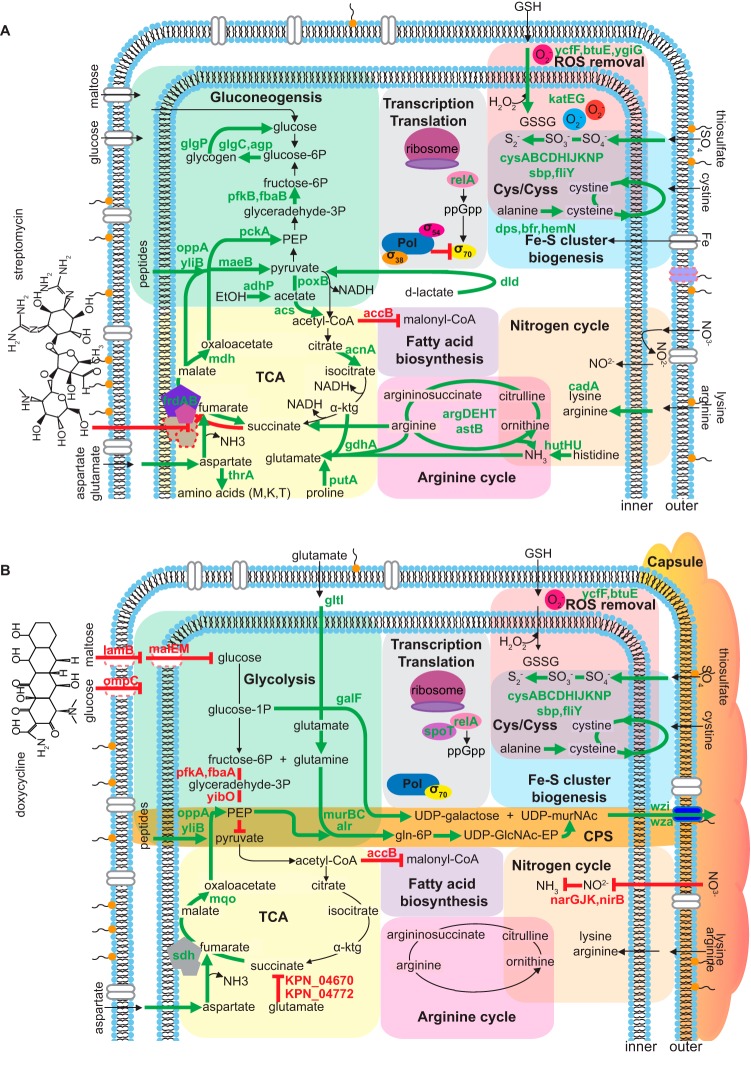
**Antibiotic-specific biochemical pathways of resistance.** Proteins that exhibited significant changes in abundance (±2-fold compared with untreated cells; *p* value < 0.05) in (*A*) streptomycin or (*B*) doxycycline-treated cells were mapped to molecular pathways by functional network associations (glycolysis, green; transcription and translation, gray; removal of reactive oxygen species (ROS), red; iron-sulfur (Fe-S) cluster biogenesis, blue; nitrogen cycle, tan; fatty acid biosynthesis, purple; arginine cycle, pink; tricarboxylic acid (TCA) cycle, yellow, capsular polysaccharide (CPS) biosynthesis, orange). Regulation of pathway reactions by proteins exhibiting unchanged (black vector), increased (green vector), or decreased (red segment) abundance. Selected cellular proteins are designated by shape: outer membrane porins (oval dimers), components involved in electron transport (pentagon), protein synthesis (oval), or removal of ROS (circle). The protein color is identical for pathway regulation that is common to both antibiotics, and subdued with red dashed borders if down-regulated in one treatment *versus* the other.

Because the culture growth curves exhibited by antibiotic-treated bacteria appeared to be unique to each antibiotic (Fig. 2*A*–2*D*), we reasoned that the proteome should display the steady-state composition of resistance, as measured by quantitative mass spectrometry (MS). We focused on the bactericidal streptomycin (SM) and bacteriostatic doxycycline (DC), which both impair protein synthesis by binding to the 16s rRNA component of the 30S ribosomal subunit, but differ in class (Table I), structure (Fig. 3), and mechanism of transport across bacterial membranes. The MS analysis of untreated and antibiotic-treated cells expanded the experimental coverage of the 5126 predicted bacterial gene products to 32% (1654), and included 614 open reading frames that encode orphan proteins predicted solely by gene annotation algorithms with no previous evidence of protein-level existence or known biochemical function (22). Antibiotic treatment resulted in substantial changes to the proteome that were reproducible across independently-cultured populations (supplemental Fig. S4 and S5), and these global effects were also highly specific to each antibiotic (Fig. 2*E*), indicating transition from the steady-state control to new proteomic trajectories. As ribosomal function is the primary target of DC and SM, treated bacteria should demonstrate alterations of the translational machinery that compensate for the toxic effects of these antibiotics. Compared with control and DC-treated bacteria, total ribosome content, quantitated as the average abundance of 54 ribosomal subunit proteins (109.3% of untreated for DC *versus* 78.1% for SM), was significantly decreased (*p* value < 0.00003) in SM-treated cells. Levels of the alarmones ppGpp and pppGpp ((p)ppGpp)), which influence protein allocation and control ribosomal synthesis (23, 24), are regulated by the activity of RelA and SpoT. RelA is recruited to stalled ribosomes where it converts GTP and ATP into pppGpp, whereas SpoT degrades ppGpp to GDP, releasing phosphate. We observed elevated levels of RelA for both DC and SM-treated cells and increased (∼2-fold) SpoT for DC-treated cells, suggesting a supportive role for alarmones in stabilizing the new proteomic trajectories. We further noted that bacteria that resisted SM, but not DC, exhibited substantially elevated levels of proteins that are transcriptionally regulated by alternative stress-response sigma factors (25) (supplemental Fig. S6, supplemental Table S2, S3). Sigma factors are multi-domain subunits of bacterial RNA polymerase that compete for binding to a limited number of holoenzymes and have essential roles in promoter recognition and initiating steps of RNA synthesis.

##### Antibiotic Transport

We hypothesized that resistance should include off-target mechanisms because the antibiotics examined must first traverse the double membrane of Gram-negative bacteria and move through the cytoplasm before reaching the ribosomal targets. Intracellular transport of SM and similar aminoglycosides involves unknown receptors and requires a proton-motive force (PMF) that is driven by the aerobic electron transport chain (26), whereas DC requires porin channels that normally accommodate sugar carbon sources (27). Therefore, we examined changes to the membrane that may limit intracellular transport of antibiotic. SM-treated bacteria exhibited elevated levels of fumarate reductase (FrdAB), which is transcriptionally activated under anaerobic conditions and reverses the tricarboxylic acid (TCA) cycle sequence from succinate to fumarate (28) (Fig. 3*A* and supplemental Table S2, S3). Furthermore, SM-treated cells had increased abundance of the lysine decarboxylase CadA, which plays a role in pH homeostasis by consuming protons and neutralizing the acidic products of carbohydrate fermentation, as well as proteins (GhrAB, TrpC, KPN_04670, KPN_04772, YhbL, Tsa) involved in the conversion of reactive electrophilic species to non-reactive small carbon compounds. The 2–4 carbon acidic intermediates can enter central metabolism and contribute to diminished aminoglycoside susceptibility by decreasing PMF and TCA activity (29). In contrast to SM treatment, DC-treated bacteria had decreased abundance of the outer-membrane porins LamB (maltose) and OmpC (glucose), as well as the proteins MalE and MalM, which are required for maltose transport across the periplasmic space (supplemental Table S2, S3). Because carbohydrate influx was likely to be dissimilar between SM- and DC-treated cells, we examined growth under aerobic or hypoxic conditions, using minimal medium with glucose as the sole carbon source. Accumulation of SM decreases under anaerobic metabolism (30), and SM-treated bacteria exhibited highly acidic culture supernatants (supplemental Fig. S7*A*) resulting from an increased fermentative utilization of glucose (supplemental Fig. S7*B*). Reduced antibiotic transport for DC-treated bacteria was a likely consequence of decreased glucose utilization (supplemental Fig. S7*B*). Collectively, these results implicated a reduction in intracellular accumulation for both antibiotics.

##### The Proteomic Transition to Resistance

To compensate for changing membrane PMF or sugar permeability, downstream proteomic remodeling of central metabolic pathways was apparent in SM and DC-treated bacteria. Aligned with reduced import of sugars, resistance to DC involved a reduction in glycolytic enzymes (Fig. 3*B* and supplemental Table S2, S3), whereas the TCA cycle was likely enhanced by the increased abundance of key enzymatic steps, mainly succinate dehydrogenase Sdh and malate dehydrogenase Mqo. In contrast to DC treatment, the increased gluconeogenesis and glycolytic enzymes observed in the SM-treated bacteria are essential for synthesis of glucose-6-phosphate from small carbon compounds as an alternative to C_6_ sugars (31, 32), and it is possible that accelerated utilization of glucose compensated for the lower energy yield that resulted from a shift to anaerobic metabolism (Fig. 3*A*; supplemental Fig. S7 and supplemental Table S2, S3). Additionally, elevated levels of phosphoenolpyruvate synthesis enzymes suggested a necessity to breakdown the accumulated byproducts of fermentative metabolism, including C_3_ and C_4_ compounds or substrates that entered central metabolism via acetyl-CoA, such as acetate, fatty acids, and ethanol (33) (Fig. 3*A* and supplemental Table S2, S3). We further observed increased prevalence of TCA cycle enzymes that do not require NAD^+^ (PoxB, AcnA, and others) (Fig. 3*A* and supplemental Table S2, S3). Preferential up-regulation of TCA reactions that do not generate NADH will result in an increased ratio of intracellular NAD^+^/NADH, potentially leading to increased tolerance to bactericidal antibiotics because of decreased superoxide generation (34). Notably, reduced hydroxyl radical formation and elimination of H_2_O_2_ induced by antibiotics was likely mediated by elevated levels of the bacterioferritin Bfr (35) and the DNA-binding protein from starved cells Dps (36, 37). Several components for turnover of arginine and histidine were also elevated in SM-treated bacteria (Fig. 3*A* and supplemental Table S2, S3). Curiously, ArgD of the arginine cycle was elevated in SM-treated cells, and this protein was also detected in tissues from infected monkeys (Fig. 1). Ammonia is the preferred nitrogen source of Gram-negative organisms, and it is possible that amino acid turnover leads to increased generation of this product (38).

The capsule of hypermucoviscous *K. pneumoniae* strains is a major virulence factor that provides protection from lethal serum factors and resistance to phagocytosis (39, 40). DC, but not SM, induced dramatically elevated levels of the capsule biosynthesis cluster (*cps*) encoded proteins GalF, Wza, and Wzi (Fig 3*B* and supplemental Table S2, S3) that are required for polysaccharide export and stabilization. The *wzi* gene is found only in bacteria that assemble capsular polysaccharide (CPS) (41), and deletion of *wzi* from capsule-producing strains of *K. pneumoniae* results in decreased bacteria-associated CPS and increased secretion of CPS into the extracellular environment (40). Based on the changes in proteins for production and efflux of capsule, we anticipated an elevated level of capsular polysaccharide in DC-treated cells. Indeed, the DC-treated bacteria exhibited 5-fold higher hypermucoviscosity compared with untreated cells (supplemental Fig. S7), and presented a mesh-like network of polymers that protruded from outer cell membranes (supplemental Fig. S8). Compared with DC-treated bacteria, CPS chains produced by untreated cells were shorter and loosely associated with the cell surface (supplemental Fig. S8). In contrast to DC- and un-treated bacteria, no CPS was detectable in SM-treated cells (supplemental Fig. S7*C*, S8*B*). Further, increased abundance of proteins encoded outside the *cps* locus that are necessary for production of CPS precursors indicated an extensive enhancement of the CPS synthesis pathway in DC-treated cells. This change included enzymes for peptidoglycan synthesis and recycling (MurBC, Alr, and others), as well as proteins for generation of phosphoenolpyruvate (Fig. 3*B*).

##### Susceptibility to Combination Antibiotics

Because resistance to SM resulted in unique biochemical pathways that were not likely to be compatible with DC resistance, we examined bacteria that were resistant to a combination of both antibiotics (SMDC). A novel proteome composition for dual antibiotic treatment was suggested by growth curves that were intermediate between those of individual drug treatment (Fig. 2*F*, supplemental Table S2, S3). SMDC-treated cells comprised protein aspects that were in common with both drugs, as well as unique signatures (Fig 2*E*; supplemental Fig. S5, S9), accounting for significant changes to 634 proteins. In all antibiotic treated cells, we observed elevated levels of proteins that link sulfur metabolism to cysteine biosynthesis and the “Cys/Cyss shuttle system” (CysABCDHIJKNP, Sbp, FliY; Fig. 3, supplemental Fig. S9) that cycles cytoplasmic cysteine and periplasmic cystine to eliminate H_2_O_2_ (42, 43). In comparing drug-specific mechanisms, SMDC-treated bacteria exhibited a decreased abundance of glucose import and utilization proteins (Gcd, OmpC, LamB, MalEM) that correlated with more aerobic utilization of glucose (supplemental Fig. S7*B*). Levels of CPS export proteins (Wza and Wzi) were lower for SMDC than DC-treated bacteria, resulting in a proportionate reduction in synthesized CPS (supplemental Fig. S7*C*, S8*D*). Specific SMDC signatures included decreased abundance of membrane proteins for the uptake of glycerol (GlpF), potassium (TrkA), and small molecules (OmpF), and increased abundance of the inner membrane proteins FtsK and ZipA, which are essential for cell division (supplemental Fig. S9). Moreover, a unique mechanism to compensate for stalled translation in the dual drug treatment was suggested by elevated levels of the ribosome biogenesis GTPase RsgA that is essential to maturation of the 30S ribosomal subunit and decreased abundance of the transcriptional repressor of methionine synthesis MetJ, which could increase initiation of translation (supplemental Fig. S9).

**Fig. 4.**
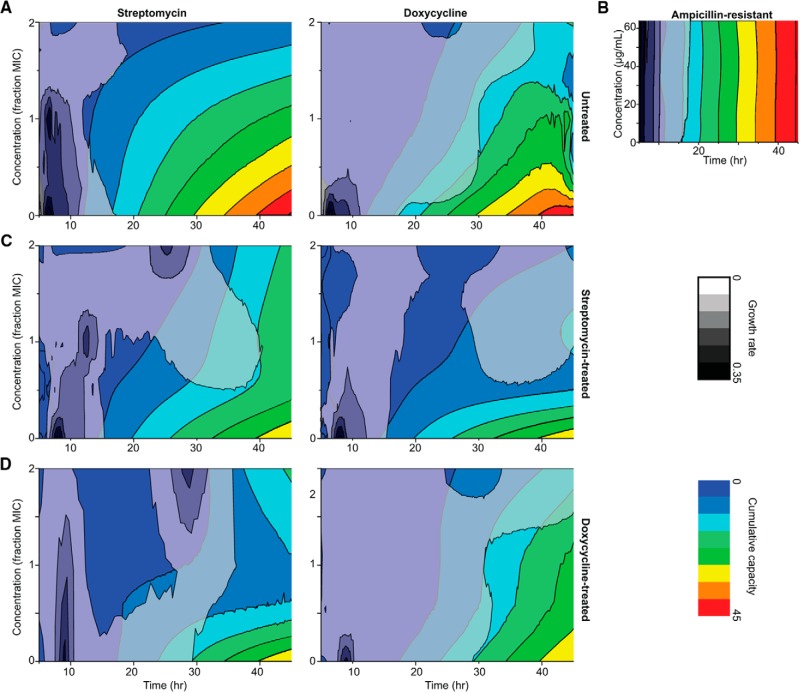
**Proteomic remodeling boosts survival during successive exposures to the same antibiotic.** Cultures of (*A*) untreated, (*B*) ampicillin resistant, (*C*) streptomycin treated, and (*D*) doxycycline treated extended spectrum β-lactamase-producing *K. pneumoniae* were treated with streptomycin (left column), doxycycline (middle column), or ampicillin (right), using 0.5–2× the minimum inhibitory concentration. Low (blue) to high (red) accumulated culture density; slow (gray) to fast (black) replication rate.

We considered the possibility that proteomic remodeling boosts the ability of bacterial populations to survive successive exposure to the same antibiotic. Simulating antibiotic use in the clinic, we examined population responses of resistant bacteria that were re-treated with antibiotics at concentrations of 0.5–2.0 MIC. Fig. 4 shows population expansion as a function of time and drug concentration. The addition of SM or DC to sensitive (untreated) cultures resulted in declining rates of replication and reduced carrying capacity that corresponded with increasing concentration of drug (Fig. 4*A*), whereas high doses of ampicillin (>60 μg/ml) did not affect cell proliferation or maximum culture density of the resistant bacteria (Fig. 4*B*). Remarkably, cultures of antibiotic-treated cells retreated with the same drug exhibited a resurgence in growth rates at concentrations > MIC that contributed to recovery of accumulated capacity with time (Fig. 4*C*, 4*D*), indicating an increase in drug-specific resistance. With SM-treatment, for example, the contour of low to high maximum culture capacity shifted vertically as the treatment dose of SM was increased (Fig. 4*C*), reflecting a shift to a higher level of resistance (Fig. 4*B*). However, we observed a horizontal collapse of SM-treated culture capacity with the addition of increasing concentrations of DC (Fig. 4*C*), indicating that sensitivity to DC was retained. Similarly, resistance to increasing concentrations of DC was exhibited by DC-treated bacteria (Fig. 4*D*), along with continued sensitivity to SM. Given the diversity of proteomic adaptations exhibited by SM- and DC-treated bacteria, it is likely that the drug-specific protective mechanisms established early in culture favored survival upon repeated exposure to the same drug. Interestingly, DC-treated cells exhibited decreased sensitivity to the highest treatment dose of SM (Fig. 4*D*), and after an extended lag phase (∼5 h) the growth curve resembled SM treatment more than DC treatment (data not shown), possibly because of the switch from DC- to SM-resistance.

## DISCUSSION

**Fig. 5.**
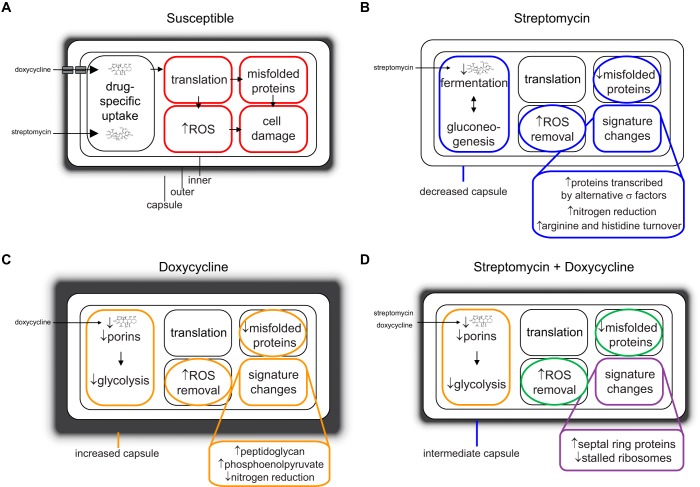
**Activated proteomic compartments of bacteria that are resistant to antibiotics targeting the ribosome.** *A*, Susceptible bacteria treated with streptomycin or doxycycline; *B*, Decreased susceptibility to streptomycin, *C*, doxycycline, or (*D*) the combination of streptomycin and doxycycline, showing mechanisms that are shared with streptomycin (blue) or doxycycline (orange), or common to both antibiotics (green).

The *K. pneumoniae* examined in our study harbors a plasmid-expressed β-lactamase that renders this bacterium resistant to most β-lactams, a class of antibiotics that contain a four-membered β-lactam ring that is structurally similar to the D-Ala-D-Ala moiety of the natural substrate (44). Growth of this ESBL isolate in response to non-carbapenem β-lactam antibiotics was identical to untreated controls (Fig. 2*A*, 2*B*), indicating that the toxic challenge was very efficiently resolved by β-lactamase activity. The antibiotics that we studied in detail have roughly the same mechanism of action but differ completely in chemical structure. SM is an aminoglycoside antibiotic that interacts with the small 16S rRNA of the 30S ribosomal subunit to inhibit binding of formyl-methionyl-tRNA (45), and the tetracycline class antibiotic DC inhibits binding of aminoacyl-tRNA to the mRNA-ribosome complex of the 30S subunit (46). The divergence between bacteria that grew in the presence of either DC or SM indicated that chemical structure was a primary driver of the global proteomic remodeling (Fig. 5). Although retreatment of bacteria with the same antibiotic produced upward shifts in MICs, antibiotic treated cultures reverted to the original drug susceptibility profiles upon removal of antibiotic and culturing with fresh media, confirming the reversible nature of the proteomic remodeling (47, 48).

Instead of focusing on the first generation of surviving bacteria (49), we examined progeny cells that grew out from antibiotic-treated populations. Random precursors should have a selective advantage over the rest of the population, and these bacteria expand in number to rapidly overtake less fit bacteria. By starting cultures with ∼200 bacteria that were synchronized in replication, we minimized the possible contribution of dormant and dying cells or chemical communication between cells. A framework was recently proposed (49) for classifying the drug response of bacterial strains by measurement of the MIC together with a quantitative indicator of tolerance, the minimum duration for killing (MDK). The reported range for tolerant precursors based on the MDK is 0.001 - 10% (50), whereas ∼1.5% of the bacterial population in our model give rise to resistant bacteria. Instead of a stable metric, it is conceivable that the frequency of precursors represents the inherent heterogeneity of the total bacterial population or the multiple distinct cellular physiologies within a population (51). Disproportionate partitioning of the proteome (52, 53) or (p)ppGpp-like signaling molecules in daughter cells during replication as a bet-hedging strategy will ensure fitness of the overall population in unstable environments.

Compared with single antibiotic efficacy, combination therapies attempt to use antibiotics with independent mechanisms of action, generally a β-lactam and an aminoglycoside. Combining two or more antibiotics as a treatment option can result in additive and synergistic activities that potentially reduce the likelihood of the emergence of resistant bacteria, whereas antagonistic effects may lower efficacy. However, selecting antibiotics for the most efficacious combination therapy is empirical (54) or based on trial and error. Adjusting the chemical properties of antibiotics to improve accumulation within Gram-negative pathogens can convert ineffective compounds into highly active antimicrobials (55). In a similar manner, proteomic adjustments of the bacteria responding to one antibiotic can alter antibiotic properties of a second compound. From our results, resistance to combination treatment with two antibiotics was mediated by both drug-specific and unique mechanisms (Fig. 5), perhaps because some components of the remodeled proteomes that were necessary for survival in the presence of a one drug may increase susceptibility to the other drug. These results further suggest that antibiotics with similar molecular targets but different chemical structures may be useful for combination therapies if used under conditions that do not promote resistance.

## DATA AVAILABILITY

The mass spectrometry proteomics data have been submitted to the ProteomeXchange Consortium via the PRIDE data repository (18) with the dataset identifiers PXD005587, PXD009175, and PXD010244. In addition, data sets can be visualized in MS-viewer (http://msviewer.ucsf.edu/prospector/cgi-bin/msform.cgi?form=msviewer) with the following search keys: lyhdoz5qdr, ljjmcsbhke, kqlulgtl0v, lyibssyves.

## Supplementary Material

Supplemental Data

Supplemental Table 2. Klebsiella pneumoniae proteins detected using MaxQuant.

Supplemental Table 3. Klebsiella pneumoniae proteins detected using Proteome Discoverer.

## Footnotes

^1^ The abbreviations used are:

*KpV513*: *Klebsiella pneumoniae V513*
CFU: colony forming units
CPS: capsular polysaccharide
Ct: time to detectable growth
DC: doxycycline
EM: electron microscopy
ESBL: extended spectrum beta-lactamase
LB: luria-bertani
MDK: minimum duration of killing
MIC: minimum inhibitory concentration
PCA: principal component analysis
PMF: proton motive force
SM: streptomycin
TCA: tricarboxylic acid.

## References

[1] Struve C., Bojer M., and Krogfelt K. A. (2008) Characterization of Klebsiella pneumoniae type 1 fimbriae by detection of phase variation during colonization and infection and impact on virulence. Infect. Immun. 76, 4055–4065 18559432 10.1128/IAI.00494-08 PMC2519443

[2] Struve C., Bojer M., and Krogfelt K. A. (2009) Identification of a conserved chromosomal region encoding Klebsiella pneumoniae type 1 and type 3 fimbriae and assessment of the role of fimbriae in pathogenicity. Infect. Immun. 77, 5016–5024 19703972 10.1128/IAI.00585-09 PMC2772557

[3] Paczosa M. K., and Mecsas J. (2016) Klebsiella pneumoniae: Going on the Offense with a Strong Defense. Microbiol. Mol. Biol. Rev. 80, 629–661 27307579 10.1128/MMBR.00078-15 PMC4981674

[4] Gu D., Dong N., Zheng Z., Lin D., Huang M., Wang L., Chan E. W., Shu L., Yu J., Zhang R., and Chen S. (2018) A fatal outbreak of ST11 carbapenem-resistant hypervirulent *Klebsiella pneumoniae* in a Chinese hospital: a molecular epidemiological study. Lancet Infectious Dis. 18, 37–46 10.1016/S1473-3099(17)30489-9 28864030

[5] Burke R. L., Whitehouse C. A., Taylor J. K., and Selby E. B. (2009) Epidemiology of invasive Klebsiella pneumoniae with hypermucoviscosity phenotype in a research colony of nonhuman primates. Comparative Med. 59, 589–597 PMC2798845 20034435

[6] Twenhafel N. A., Whitehouse C. A., Stevens E. L., Hottel H. E., Foster C. D., Gamble S., Abbott S., Janda J. M., Kreiselmeier N., and Steele K. E. (2008) Multisystemic abscesses in African green monkeys (Chlorocebus aethiops) with invasive Klebsiella pneumoniae–identification of the hypermucoviscosity phenotype. Veterinary Pathol. 45, 226–231 10.1354/vp.45-2-226 18424839

[7] Blango M. G., and Mulvey M. A. (2010) Persistence of uropathogenic Escherichia coli in the face of multiple antibiotics. Antimicrob Agents Chemother 54, 1855–1863 20231390 10.1128/AAC.00014-10 PMC2863638

[8] Claudi B., Spröte P., Chirkova A., Personnic N., Zankl J., Schürmann N., Schmidt A., and Bumann D. (2014) Phenotypic variation of Salmonella in host tissues delays eradication by antimicrobial chemotherapy. Cell 158, 722–733 25126781 10.1016/j.cell.2014.06.045

[9] Livermore D. M. (2009) Has the era of untreatable infections arrived? J. Antimicrob. Chemother. 64, i29–i36 19675016 10.1093/jac/dkp255

[10] Amato S. M., Orman M. A., and Brynildsen M. P. (2013) Metabolic control of persister formation in Escherichia coli. Mol. Cell 50, 475–487 23665232 10.1016/j.molcel.2013.04.002

[11] Shan Y., Brown Gandt A., Rowe S. E., Deisinger J. P., Conlon B. P., and Lewis K. (2017) ATP-Dependent Persister Formation in Escherichia coli. MBio 8 10.1128/mBio.02267-16 PMC5296605 28174313

[12] Andrews J. M. (2001) Determination of minimum inhibitory concentrations. J. Antimicrob. Chemother. 48, 5–16 11420333 10.1093/jac/48.suppl_1.5

[13] Fang C. T., Chuang Y. P., Shun C. T., Chang S. C., and Wang J. T. (2004) A novel virulence gene in Klebsiella pneumoniae strains causing primary liver abscess and septic metastatic complications. J. Exp. Med. 199, 697–705 14993253 10.1084/jem.20030857 PMC2213305

[14] Wisniewski J. R., Zougman A., Nagaraj N., and Mann M. (2009) Universal sample preparation method for proteome analysis. Nat. Methods 6, 359–362 19377485 10.1038/nmeth.1322

[15] Rappsilber J., Mann M., and Ishihama Y. (2007) Protocol for micro-purification, enrichment, pre-fractionation and storage of peptides for proteomics using StageTips. Nat. Protoc. 2, 1896–1906 17703201 10.1038/nprot.2007.261

[16] Lazar C., Gatto L., Ferro M., Bruley C., and Burger T. (2016) Accounting for the multiple natures of missing values in label-free quantitative proteomics data sets to compare imputation strategies. J. Proteome Res. 15, 1116–1125 26906401 10.1021/acs.jproteome.5b00981

[17] Webb-Robertson B. J., Wiberg H. K., Matzke M. M., Brown J. N., Wang J., McDermott J. E., Smith R. D., Rodland K. D., Metz T. O., Pounds J. G., and Waters K. M. (2015) Review, evaluation, and discussion of the challenges of missing value imputation for mass spectrometry-based label-free global proteomics. J. Proteome Res. 14, 1993–2001 25855118 10.1021/pr501138h PMC4776766

[18] Vizcaino J. A., Deutsch E. W., Wang R., Csordas A., Reisinger F., Ríos D., Dianes J. A., Sun Z., Farrah T., Bandeira N., Binz P. A., Xenarios I., Eisenacher M., Mayer G., Gatto L., Campos A., Chalkley R. J., Kraus H. J., Albar J. P., Martinez-Bartolomé S., Apweiler R., Omenn G. S., Martens L., Jones A. R., and Hermjakob H. (2014) ProteomeXchange provides globally coordinated proteomics data submission and dissemination. Nat. Biotechnol. 32, 223–226 24727771 10.1038/nbt.2839 PMC3986813

[19] Holt K. E., Wertheim H., Zadoks R. N., Baker S., Whitehouse C. A., Dance D., Jenney A., Connor T. R., Hsu L. Y., Severin J., Brisse S., Cao H., Wilksch J., Gorrie C., Schultz M. B., Edwards D. J., Nguyen K. V., Nguyen T. V., Dao T. T., Mensink M., Minh V. L., Nhu N. T., Schultsz C., Kuntaman K., Newton P. N., Moore C. E., Strugnell R. A., and Thomson N. R. (2015) Genomic analysis of diversity, population structure, virulence, and antimicrobial resistance in Klebsiella pneumoniae, an urgent threat to public health. Proc. Natl. Acad. Sci. U.S.A. 112, E3574–E3581 26100894 10.1073/pnas.1501049112 PMC4500264

[20] Johnson A. S. A., Madden K. M., and Şahin AA. (2017) Discovering Discrete Dynamical Systems (MATHEMATICAL ASSN AMERICA).

[21] Reding-Roman C., Reding-Roman C., Hewlett M., Duxbury S., Gori F., Gudelj I., and Beardmore R. (2017) The unconstrained evolution of fast and efficient antibiotic-resistant bacterial genomes. Nat. Ecol. Evol. 1, 50 28812723 10.1038/s41559-016-0050

[22] Suh M. J., Keasey S. L., Brueggemann E. E., and Ulrich R. G. (2017) Antibiotic-dependent perturbations of extended spectrum beta-lactamase producing Klebsiella pneumoniae proteome. Proteomics 17 10.1002/pmic.201700003 28198105

[23] Potrykus K and Cashel M. (2008) (p) ppGpp: still magical? Annu. Rev. Microbiol. 62, 35–51 18454629 10.1146/annurev.micro.62.081307.162903

[24] Lemke J. J., et al (2011) Direct regulation of Escherichia coli ribosomal protein promoters by the transcription factors ppGpp and DksA. Proc. Natl. Acad. Sci. U.S.A. 108, 5712–5717 21402902 10.1073/pnas.1019383108 PMC3078377

[25] Seo J. H., Hong J. S., Kim D., Cho B. K., Huang T. W., Tsai S.F., Palsson B. O., and Charusanti P. (2012) Multiple-omic data analysis of Klebsiella pneumoniae MGH 78578 reveals its transcriptional architecture and regulatory features. BMC Genomics 13, 679 23194155 10.1186/1471-2164-13-679 PMC3536570

[26] Taber H. W., Mueller J. P., Miller P. F., and Arrow A. S. (1987) Bacterial uptake of aminoglycoside antibiotics. Microbiol. Rev. 51, 439–457 3325794 10.1128/mr.51.4.439-457.1987 PMC373126

[27] Chopra I., and Roberts M. (2001) Tetracycline antibiotics: mode of action, applications, molecular biology, and epidemiology of bacterial resistance. Microbiol. Mol. Biol. Rev. 65, 232–260; second page, table of contents. 11381101 10.1128/MMBR.65.2.232-260.2001 PMC99026

[28] Cecchini G., Schröder I., Gunsalus R. P., and Maklashina E. (2002) Succinate dehydrogenase and fumarate reductase from Escherichia coli. Biochim. Biophys. Acta (BBA) - Bioenergetics 1553, 140–157 11803023 10.1016/s0005-2728(01)00238-9

[29] Meylan S., Porter C. B. M., Yang J. H., Belenky P., Gutierrez A., Lobritz M. A., Park J., Kim S. H., Moskowitz S. M., and Collins J. J. (2017) Carbon sources tune antibiotic susceptibility in Pseudomonas aeruginosa via tricarboxylic acid cycle control. Cell Chem. Biol. 24, 195–206 28111098 10.1016/j.chembiol.2016.12.015 PMC5426816

[30] Kogut M., Lightbrown J. W., and Isaacson P. (1965) Streptomycin Action and Anaerobiosis. J. Gen. Microbiol. 39, 155–164 14324962 10.1099/00221287-39-2-155

[31] Bologna F. P., Andreo C. S., and Drincovich M. F. (2007) Escherichia coli malic enzymes: two isoforms with substantial differences in kinetic properties, metabolic regulation, and structure. J. Bacterio.l 189, 5937–5946 10.1128/JB.00428-07 PMC1952036 17557829

[32] Eydallin G., Montero M., Almagro G., Sesma M. T., Viale A. M., Muñoz F. J., Rahimpour M., Baroja-Fernández E., and Pozueta-Romero J. (2010) Genome-wide screening of genes whose enhanced expression affects glycogen accumulation in Escherichia coli. DNA Res. 17, 61–71 20118147 10.1093/dnares/dsp028 PMC2853380

[33] Spaans S. K., Weusthuis R. A., van der Oost J., and Kengen S. W. (2015) NADPH-generating systems in bacteria and archaea. Front. Microbiol. 6, 742 26284036 10.3389/fmicb.2015.00742 PMC4518329

[34] Kohanski M. A., Dwyer D. J., Hayete B., Lawrence C. A., and Collins J. J. (2007) A common mechanism of cellular death induced by bactericidal antibiotics. Cell 130, 797–810 17803904 10.1016/j.cell.2007.06.049

[35] Crow A., Lawson T. L., Lewin A., Moore G. R., and Le Brun N. E. (2009) Structural basis for iron mineralization by bacterioferritin. J. Am. Chem. Soc. 131, 6808–6813 19391621 10.1021/ja8093444

[36] Bradley J. M., Svistunenko D. A., Lawson T. L., Hemmings A. M., Moore G. R., and Le Brun N. E. (2015) Three aromatic residues are required for electron transfer during iron mineralization in bacterioferritin. Angew Chem. Int. Ed. Engl. 54, 14763–14767 26474305 10.1002/anie.201507486 PMC4691338

[37] Bellapadrona G., Ardini M., Ceci P., Stefanini S., and Chiancone E. (2010) Dps proteins prevent Fenton-mediated oxidative damage by trapping hydroxyl radicals within the protein shell. Free Radic. Biol. Med. 48, 292–297 19892013 10.1016/j.freeradbiomed.2009.10.053

[38] Bender R. A. (2010) A NAC for regulating metabolism: the nitrogen assimilation control protein (NAC) from Klebsiella pneumoniae. J. Bacteriol. 192, 4801–4811 20675498 10.1128/JB.00266-10 PMC2944532

[39] Hsu C. R., Lin T. L., Chen Y. C., Chou H. C., and Wang J. T. (2011) The role of Klebsiella pneumoniae rmpA in capsular polysaccharide synthesis and virulence revisited. Microbiology 157, 3446–3457 21964731 10.1099/mic.0.050336-0

[40] Alvarez D., Merino S., Tomas J. M., Benedi V. J., and Alberti S. (2000) Capsular polysaccharide is a major complement resistance factor in lipopolysaccharide O side chain-deficient Klebsiella pneumoniae clinical isolates. Infect. Immun. 68, 953–955 10639470 10.1128/iai.68.2.953-955.2000 PMC97229

[41] Bushell S. R., Mainprize I. L., Wear M. A., Lou H., Whitfield C., and Naismith J. H. (2013) Wzi is an outer membrane lectin that underpins group 1 capsule assembly in Escherichia coli. Structure 21, 844–853 23623732 10.1016/j.str.2013.03.010 PMC3791409

[42] Ohtsu I., Kawano Y., Suzuki M., Morigasaki S., Saiki K., Yamazaki S., Nonaka G., and Takagi H. (2015) Uptake of L-cystine via an ABC transporter contributes defense of oxidative stress in the L-cystine export-dependent manner in Escherichia coli. PLoS ONE 10, e0120619 25837721 10.1371/journal.pone.0120619 PMC4383340

[43] Ohtsu I., Wiriyathanawudhiwong N., Morigasaki S., Nakatani T., Kadokura H., and Takagi H. (2010) The L-cysteine/L-cystine shuttle system provides reducing equivalents to the periplasm in Escherichia coli. J. Biol. Chem. 285, 17479–17487 20351115 10.1074/jbc.M109.081356 PMC2878512

[44] Tipper D. J., and Strominger J. L. (1965) Mechanism of action of penicillins: a proposal based on their structural similarity to acyl-D-alanyl-D-alanine. Proc. Natl. Acad. Sci. U.S.A. 54, 1133–1141 5219821 10.1073/pnas.54.4.1133 PMC219812

[45] Sharma D., Cukras A. R., Rogers E. J., Southworth D. R., and Green R. (2007) Mutational analysis of S12 protein and implications for the accuracy of decoding by the ribosome. J. Mol. Biol. 374, 1065–1076 17967466 10.1016/j.jmb.2007.10.003 PMC2200631

[46] Geigenmuller. U., and Nierhaus K. H. (1986) Tetracycline can inhibit tRNA binding to the ribosomal P site as well as to the A site. Eur. J. Biochem. 161, 723–726 3641718 10.1111/j.1432-1033.1986.tb10499.x

[47] Dou H., Jiang M., Peng H., Chen D., and Hong Y. (2003) pH-dependent self-assembly: micellization and micelle-hollow-sphere transition of cellulose-based copolymers. Angew Chem. In.t Ed. Engl. 42, 1516–1519 10.1002/anie.200250254 12698488

[48] Lewis K. (2010) Persister cells. Annu. Rev. Microbiol. 64, 357–372 20528688 10.1146/annurev.micro.112408.134306

[49] Balaban N. Q., Merrin J., Chait R., Kowalik L., and Leibler S. (2004) Bacterial persistence as a phenotypic switch. Science 305, 1622–1625 15308767 10.1126/science.1099390

[50] Hofsteenge N., van Nimwegen E., and Silander O. K. (2013) Quantitative analysis of persister fractions suggests different mechanisms of formation among environmental isolates of E. coli. BMC Microbiol. 13, 25 23379956 10.1186/1471-2180-13-25 PMC3682893

[51] Allison K. R., Brynildsen M. P., and Collins J. J. (2011) Heterogeneous bacterial persisters and engineering approaches to eliminate them. Curr. Opin. Microbiol. 14, 593–598 21937262 10.1016/j.mib.2011.09.002 PMC3196368

[52] Bergmiller T., Andersson A. M. C., Tomasek K., Balleza E., Kiviet D. J., Hauschild R., Tkaèik G., and Guet C. C. (2017) Biased partitioning of the multidrug efflux pump AcrAB-TolC underlies long-lived phenotypic heterogeneity. Science 356, 311–315 28428424 10.1126/science.aaf4762

[53] Rego E. H., Audette R. E., and Rubin E. J. (2017) Deletion of a mycobacterial divisome factor collapses single-cell phenotypic heterogeneity. Nature 546, 153–157 28569798 10.1038/nature22361 PMC5567998

[54] Kumar A., Zarychanski R., Light B., Parrillo J., Maki D., Simon D., Laporta D., Lapinsky S., Ellis P., Mirzanejad Y., Martinka G., Keenan S., Wood G., Arabi Y., Feinstein D., Kumar A., Dodek P., and Kravetsky L. (2010) Early combination antibiotic therapy yields improved survival compared with monotherapy in septic shock: a propensity-matched analysis. Crit. Care Med. 38, 1773–1785 20639750 10.1097/CCM.0b013e3181eb3ccd

[55] Richter M. F., Drown B. S., Riley A. P., Garcia A., Shirai T., Svec R. L., and Hergenrother P. J. (2017) Predictive compound accumulation rules yield a broad-spectrum antibiotic. Nature 545, 299–304 28489819 10.1038/nature22308 PMC5737020

