# Supplementary material for: Decreased Antibiotic Susceptibility Driven by Global Remodeling of the Klebsiella pneumoniae Proteome
Source: Mol Cell Proteomics. 2019 Jan 7;18(4):657–68. doi: 10.1074/mcp.RA118.000739 (PMC6442359; doi:10.1074/mcp.RA118.000739)
Supplement: Supplemental Data [file 136688_3_supp_262792_pktvqm.pdf]

Supplemental Data

Supplemental Table S1. Antibiotic susceptibility of Gram-negative bacteria.

		Minimum inhibitory concentration (MIC, µg/mL)				
Bacteria	CFU/mL*	Streptomycin	Ampicillin	Doxycycline	Colistin	Ciprofloxacin
K. pneumoniae V513 (NHP)	$1.16x10^9$	4	>128	1	2	ND
K. pneumoniae (human)	$1.02x10^9$	2	>128	1	2	4
Escherichia coli	$1.37x10^9$	128	>128	64	64	32
Acinetobacter baumannii	$1.18x10^9$	2	8	<0.25	2	<0.25
Yersinia pestis	$0.78x10^9$	8	ND	1	ND	ND

*Colony forming units (CFU), SEM < 15% (n=3)
ND, not determined

Supplemental Table S2. Klebsiella pneumoniae proteins quantified using MaxQuant software. Refer to additional .xls file.

Supplemental Table S3. Klebsiella pneumoniae proteins identified using Proteome Discoverer software. Refer to additional .xls file.

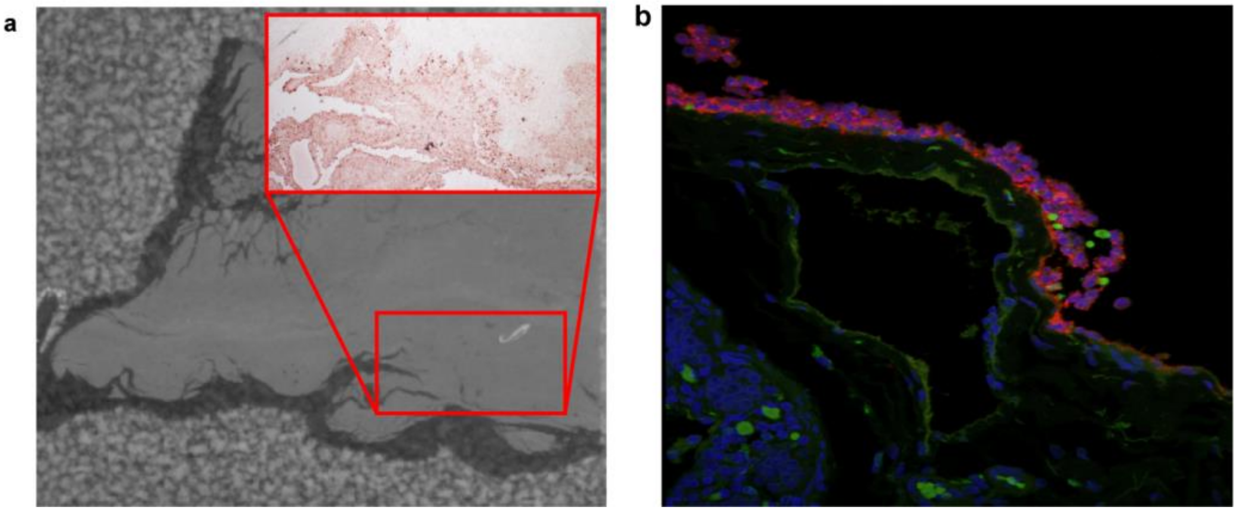

Supplemental Figure S1. *Klebsiella pneumoniae* infection in the large intestine of a *C. aethiops sabaeus* monkey. Evaluation of cecum tissue procured during necropsy of a *C. a. sabaeus* monkey that presented multiple abscesses of the gastrointestinal tract and succumbed to *K. pneumoniae* infection. (**a**) Histological image and immunohistochemistry (inset) of infected cecum tissue. Immunohistochemistry was performed using a *K. pneumoniae*-specific antibody to visualize bacteria (brown). (**b**) Immunofluorescence image of infected cecum tissue (nuclei (blue), macrophages (green), and *K. pneumoniae* cells (red)).

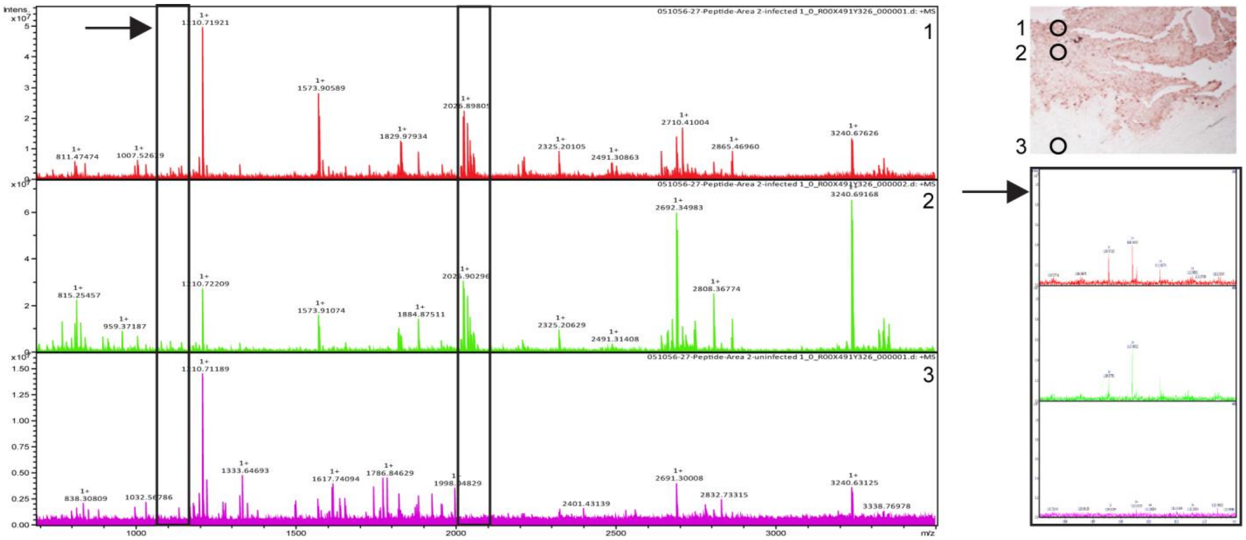

Supplemental Figure S2. Mass spectrometry-imaging of *Klebsiella pneumoniae* infected tissues. MALDI mass spectra (m/z 700 – 3500, left) of MALDI FT-ICR-MS from cecum tissue (top right) that exhibited significant bacterial burden (1,2) by immunohistochemistry using a *K. pneumoniae*-specific antibody, or adjacent uninfected tissue (3). Black boxes indicate unique m/z values observed only in infected areas, and the arrow designates spectra that are enlarged (right bottom) for clarity purposes. Spectra in positive ion mode were generated using 100 laser shots at a frequency of 1 kHz.

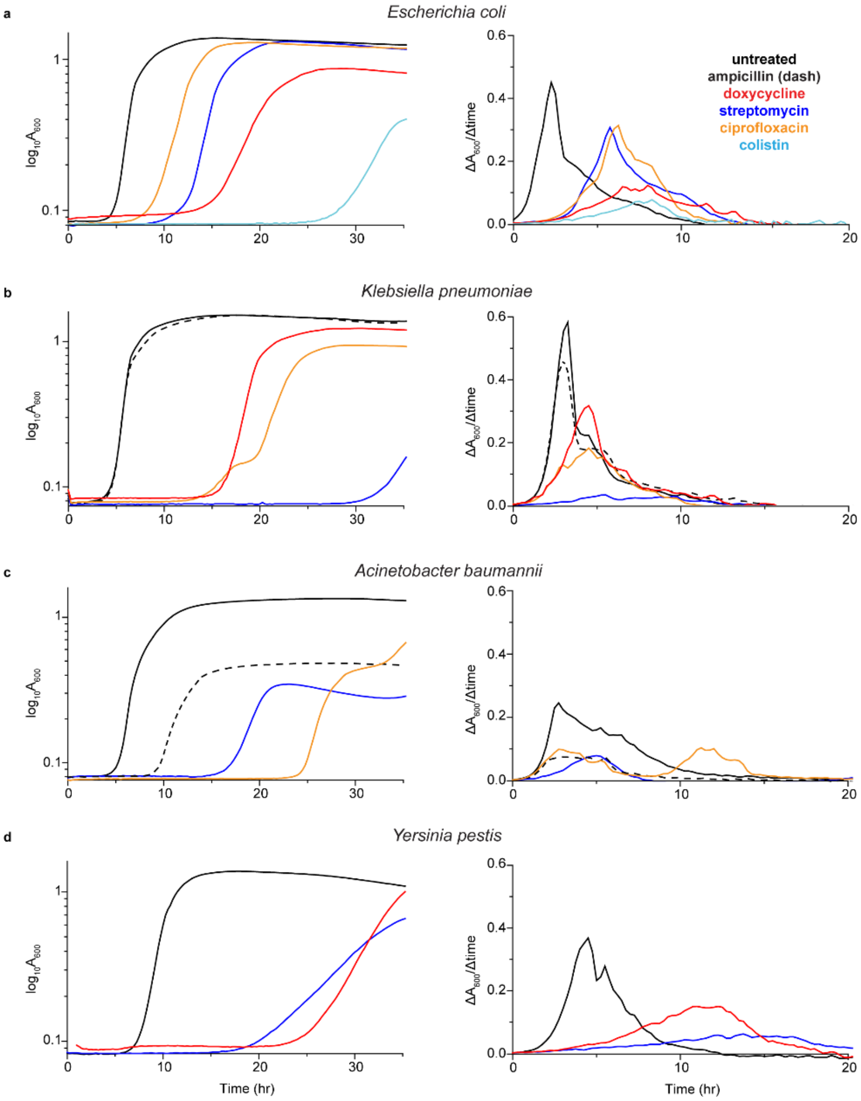

Supplemental Figure S3. Growth of Gram-negative pathogens in response to antibiotics. Clinical isolates from human sepsis infections caused by *Escherichia coli* (**a**), *Klebsiella pneumoniae* (**b**), or *Acinetobacter baumannii* (**c**), and an attenuated lab strain of *Yersinia pestis* (**d**) were cultured untreated (black) or in the presence of susceptible antibiotics at the MIC_{50}. Culture growth curves (left) and rates of replication (right) based on first derivative growth curves, overlaid from the time to detectable increases in absorbance, are shown.

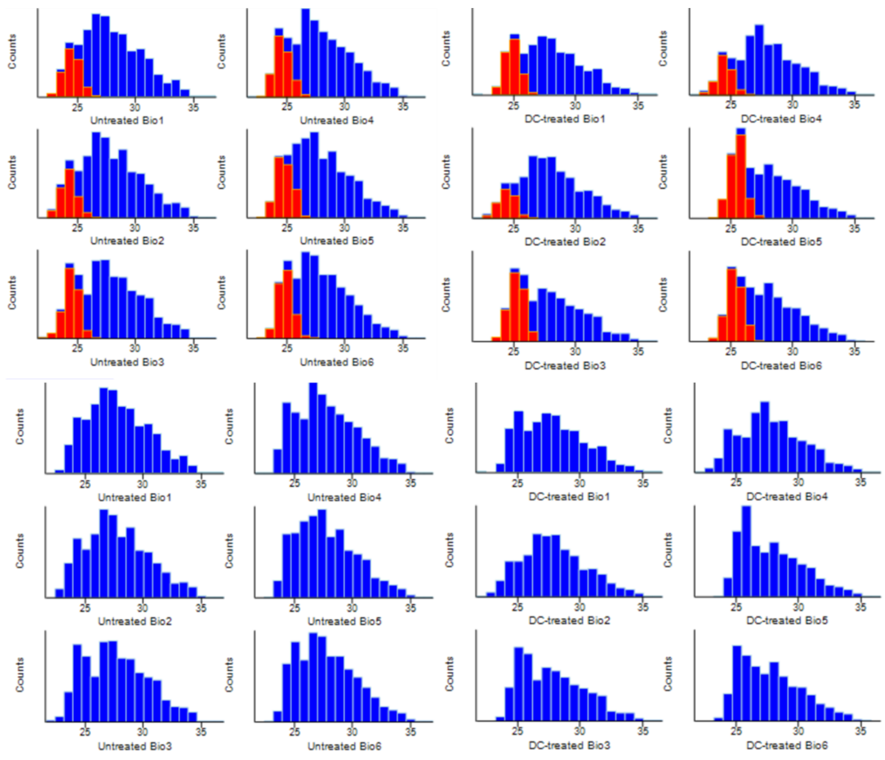

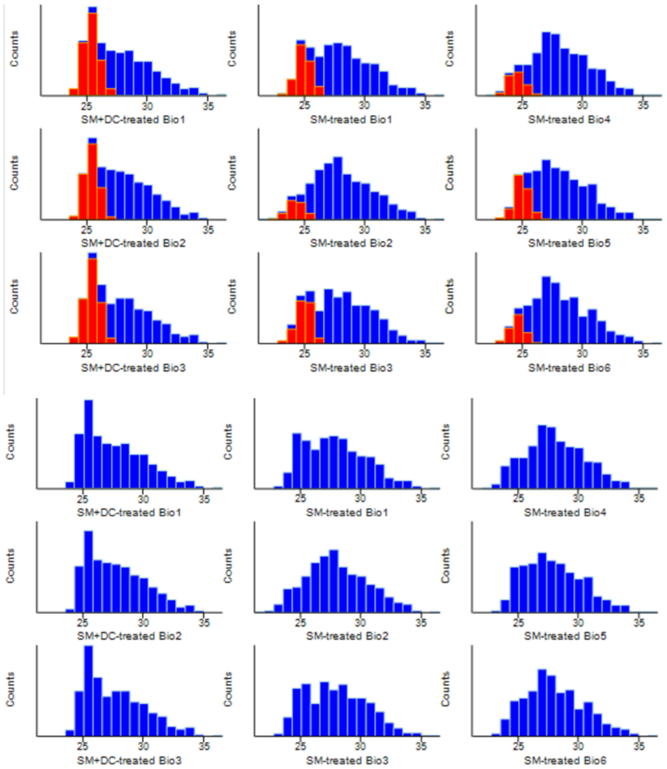

Supplemental Figure S4: Histogram generated using log transformed expression values of identified proteins before (blue) and after (red) applying imputation algorithm (16, 17). For each replicate, observed proteins were first sorted from high to low abundance. Because missing values represent low abundance measurements, we calculated the mean and standard deviation of measured values of the 25% least abundant proteins. Next, we randomly sampled the normal distribution of lowest abundance proteins to extract positive numbers for MV imputation. Finally, we repeated the imputation processes 3 times and considered the average as the final result.

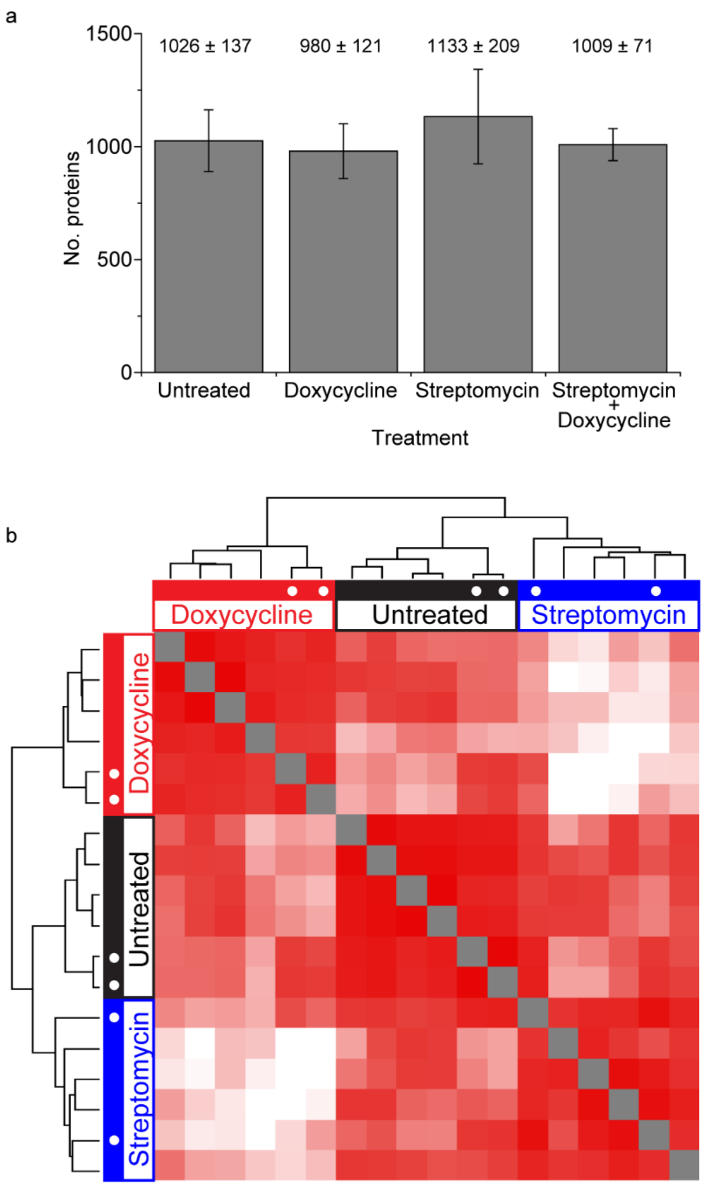

Supplemental Figure S5. *Klebsiella pneumoniae* proteins detected by shotgun proteomics. (**a**) Number of *K. pneumoniae* proteins (± standard deviation) identified from each treatment condition (n = 3 for Streptomycin + Doxycycline; n = 6 for all others). (**b**) Reproducibility of proteomes by Pearson correlation coefficient gradient, colored from 0.78 (white) to 1 (red), across independently-cultured populations (untreated, black; streptomycin, blue; doxycycline, red) and two mass spectrometry platforms (Fusion, white dot; Elite, all others). Correlation is based on >1,000 *K. pneumoniae* proteins.

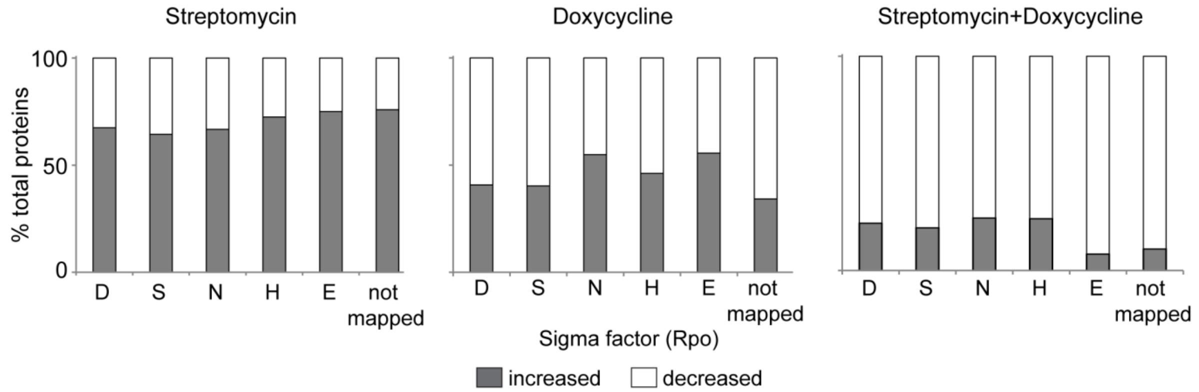

Supplemental Figure S6. Streptomycin-treated bacteria exhibit elevated levels of proteins that are transcriptionally regulated by alternative sigma factors. Proteins that exhibited significant changes in abundance (±2-fold compared to untreated cells, p-value < 0.05) in streptomycin (left), doxycycline (middle), or streptomycin+doxycycline (right) treated cells were mapped to sigma factor (Rpo) sites identified by Seo *et al.*(23)

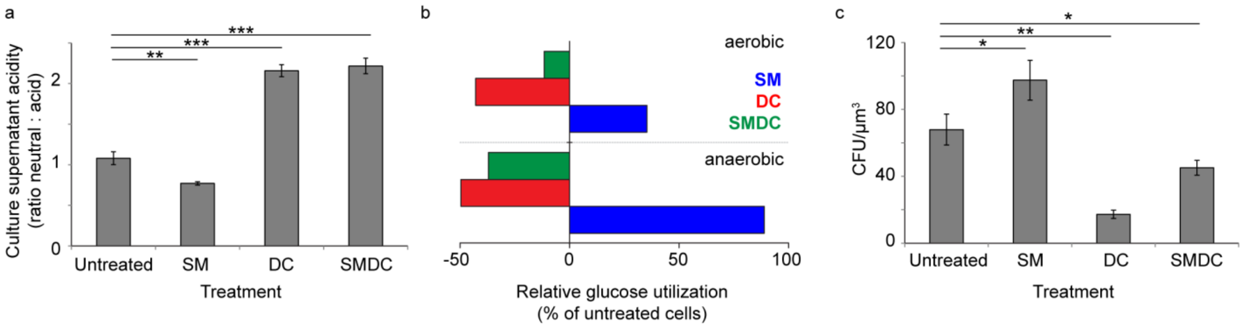

Supplemental Figure S7. Metabolic activity and capsule synthesis of antibiotic-treated bacteria. *K. pneumoniae* that were cultured untreated, or with streptomycin (SM), doxycycline (DC), or a combination of both antibiotics (SMDC) at the MIC_{50} were harvested during mid-exponential growth. (**a**) Bromothymol blue was added to culture supernatants ($n = 3$) and absorbance at 620nm (blue, $pH \geq 7.2$) and 425nm (yellow, $pH \leq 6$) was measured. Culture supernatant acidity is shown as a ratio of absorbance at 620nm : 425nm. (**b**) Cell pellets were washed twice in antibiotic-free broth and inoculated as stabs into media containing only glucose as a carbohydrate source and bromothymol blue as an indicator of acid production. Glucose utilization under hypoxic (strong acid production, anaerobic) or aerobic (weak acid production) conditions was measured after 5 hrs. Values shown are levels of acid production from glucose metabolism relative to untreated control bacteria. (**c**) Capsule production was evaluated by cell pellet volume ($n = 4$) per colony forming units (CFU) per μm^3, ± standard deviation. Treatments that were statistically different from untreated control are indicated (* $p < 0.03$, ** $p < 0.005$, *** $p < 0.0001$; Student's two-tailed t-test).

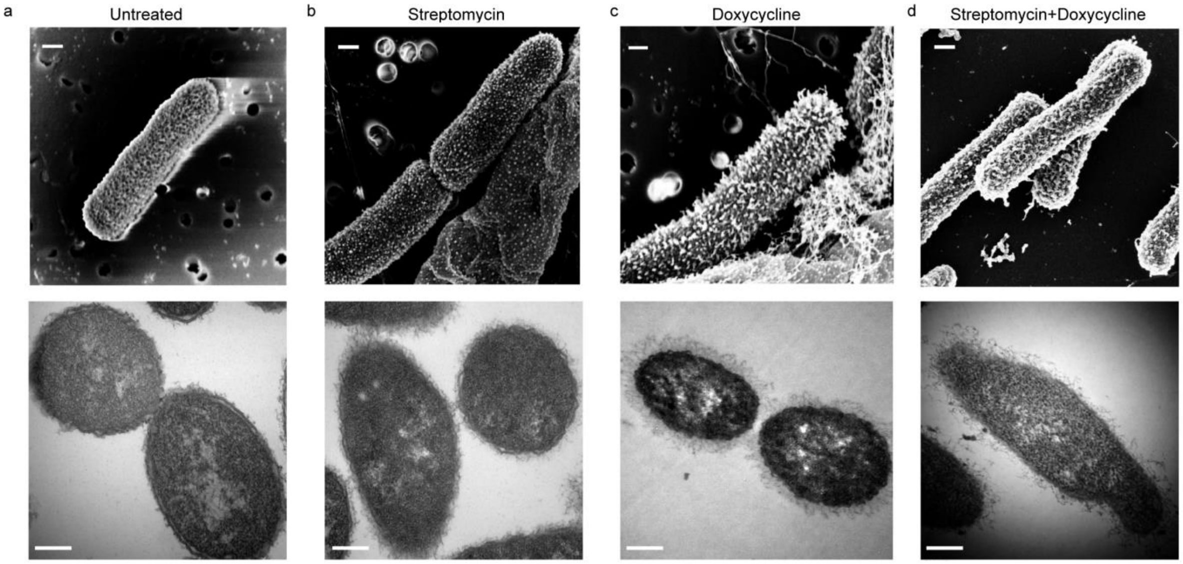

Supplemental Figure S8. Increased hypermucoviscosity is specific to doxycycline-treated bacteria. Electron micrographs of (**a**) untreated *K. pneumoniae*, and bacteria treated with (**b**) streptomycin, (**c**) doxycycline, or (**d**) a combination of streptomycin and doxycycline. Top row, scanning electron micrographs; bottom row, transmission electron micrographs. Scale bars represent 200nm for each panel.

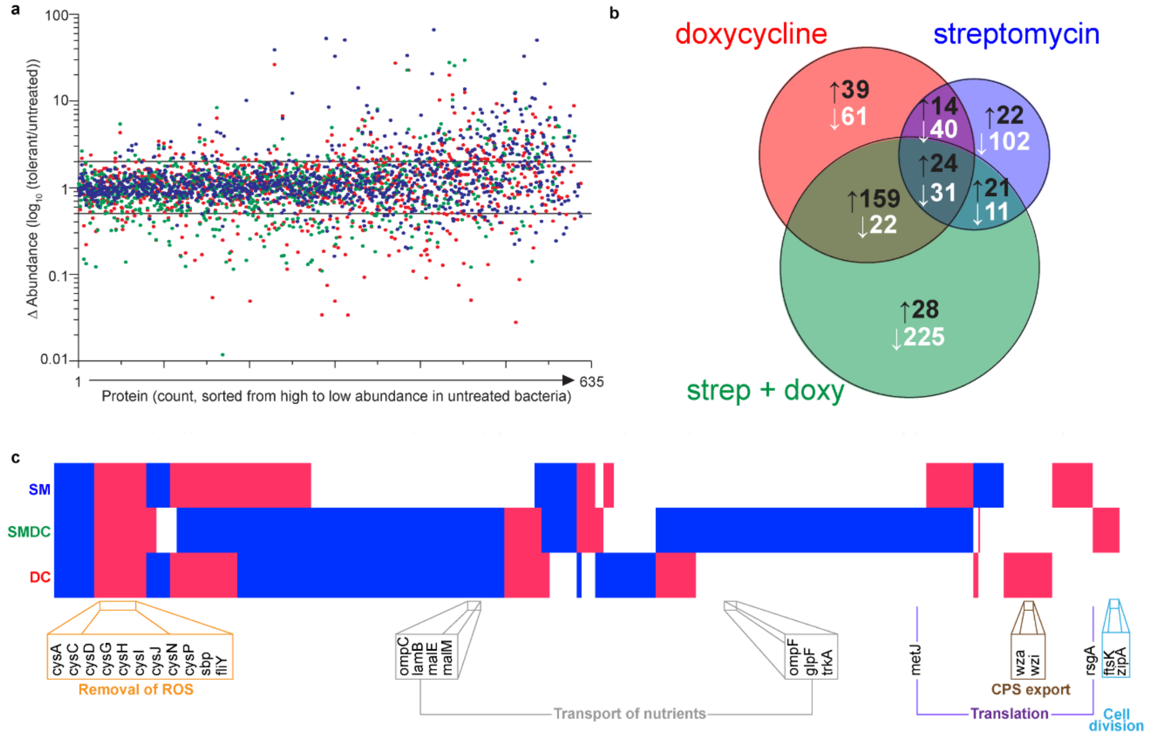

Supplemental Figure S9. Drug-specific changes to the proteomes of antibiotic-treated bacteria. *Klebsiella pneumoniae* proteins that exhibited significant changes in abundance (±2-fold compared to untreated cells, p-value < 0.05) in doxycycline (red) or streptomycin (blue) treated cells, or bacteria that resisted a dual treatment of streptomycin + doxycycline (green). (**a**) Relative abundance of 634 proteins that were detected in at least one antibiotic-treated cell type. Proteins are sorted from high to low abundance, based on spectral counts in untreated cells. (**b**) Venn diagram of common, shared, and unique proteins. Numbers in white reflect decreased abundance, black were increased in abundance. (**c**) Heat map of 634 *K. pneumoniae* proteins that exhibited significant changes in abundance in at least one cell type (SM, DC, or SM+DC) compared to untreated cells. For clarity, changes to abundance were simplified to a binary classification (decreased abundance, blue; increased abundance, red). Distinct patterns of common, shared, and unique proteins are evident, and proteins that are discussed in the text are colored by pathway.
